# So flexibel wie möglich?! Visionen für ein Studium (Sozial‑)Pädagogik 2035

**DOI:** 10.1007/s12054-022-00488-x

**Published:** 2022-05-10

**Authors:** Dorothee Kochskämper, Anna Lips

**Affiliations:** Hildesheim, Deutschland

**Keywords:** Studium, Corona, Zukunft, Befragung

## Abstract

Die Maßnahmen zur Eindämmung der Corona-Pandemie haben das Studieren und die Studienorganisation massiv verändert. Doch: Wie nachhaltig werden und sollen diese Veränderungen sein? Was wünschen sich Studierende? Der vorliegende Beitrag beschäftigt sich anhand der Daten der bundesweiten Studierendenbefragung Stu.diCo II (*N* = 2527) aus dem Sommersemester 2021 mit der Frage, was von den Studierenden während der vorrangig online stattgefundenen Semester besonders vermisst wurde und was aus Sicht der Studierenden aus den Formaten an der Hochschule in Zukunft beibehalten werden sollte. Der Fokus wird dabei auf Studierende der (Sozial‑)Pädagogik und Erziehungs- und Bildungswissenschaften (*n* = 548) gerichtet. Es wird die Frage gestellt, was sich aus den Ergebnissen der bundesweiten Studierendenbefragung Stu.diCo II für den „Blick in die Zukunft“ entnehmen lässt und Visionen für ein „Studium 2035 der Sozialen Arbeit“ entworfen.

Studieren 2035. Als Ergebnis der Nach-Corona-Reformbewegung kennzeichnet das Studieren heute in erster Linie eines: Flexibilität auf allen Ebenen. Weit weg von den ehemals starren Strukturen, schafft Hochschule größtmöglichen Raum für individuelle Bildungswege.

Lehrveranstaltungen werden hybrid, d. h. gleichzeitig in Präsenz und online, angeboten und zudem für eine mögliche spätere Abrufbarkeit aufgenommen. Die Wahl des Lehrformates wird Veranstaltung für Veranstaltung von der studierenden Person getroffen und kann den individuellen Bedürfnissen sowie der eigenen Lebenssituation (Wohnort, Arbeit, Familie, Gesundheit, etc.) angepasst werden. Studieren ist damit für eine breite Zielgruppe attraktiv und realisierbar. Dabei sind sowohl die Präsenz-, als auch die digitale sowie die aufgezeichnete Variante technisch, methodisch und didaktisch gut umgesetzt. Verzerrte oder hängende Bilder? Zitternder, abgehackter Ton? Verzögerungen? Erinnerungen der Vergangenheit! Hochschulen und Studierende sind mit den entsprechenden digitalen Endgeräten, der passenden Software sowie dauerhaft stabilen Internetverbindungen ausgestattet.

Sämtliche digitale Tools (Zoom, Teams, Mentimeter, etc.) stehen allen Hochschulangehörigen open source zur Verfügung und können somit auch von den Studierenden für Vernetzungszwecke, wie z. B. Gruppenarbeiten, genutzt werden. Inhaltlicher Austausch zwischen Lehrenden und Studierenden sowie die Vernetzung unter Studierenden findet, neben digitalen Formaten, auch vor Ort in den Hochschulen als offenes Angebot statt. Internationale Diskurse und Vernetzung gehören zum Studierendenalltag und ermöglichen vielfältige Kooperation, da Gastreferent_innen als externe Expert_innen unabhängig von Raum und Zeit regelmäßig flexibel hinzugeschaltet werden.

Aber nicht nur die Lehre, auch die Studienorganisation und -bedingungen, zeichnen sich durch eine flexible Struktur aus. Prüfungsanmeldungen, -durchführungen sowie -abgaben sind ebenso in Präsenz wie digital möglich, auch die Prüfungsformen haben neue Formate angenommen. Sprechstunden von Lehrenden und Hochschulangeboten (z. B. Studierendenberatung) sind sowohl analog als auch digital möglich. Und auch das Angebot der Bibliotheken ist sowohl vor Ort als auch digital abrufbar, wobei das digitale Angebot eine hochschulübergreifende, internationale Datenbank mit wissenschaftlicher (open source) Literatur ist, die für alle Studierenden zur Verfügung steht.

Studienpraktische Elemente und Lehrveranstaltungen sind individuell aufeinander beziehbar, sie können gleichzeitig oder nacheinander realisiert werden und sind im Verlauf des gesamten Studiums in passgenauer individueller Abstimmung umsetzbar. Hochschule ist zudem ein Ort der Begegnung und des sozialen Miteinanders. Regelmäßige Aufeinandertreffen von Studierenden untereinander sowie auch von Lehrenden und Studierenden sind ein zentraler Aspekt des Studierens. Treffen finden dabei sowohl für formalen Austausch als auch informell statt. Es wird gemeinsam gearbeitet, gelernt, diskutiert, gegessen, sich gegenseitig unterstützt. Die Möglichkeiten selbst bestimmen zu können, kennzeichnen die Studiengestaltung in hohem Maße. Die Wahl zwischen Präsenz und digital wird individuell, je nach eigener Vorstellung, getroffen.

## Was zuvor geschah

Frühjahr 2020: Die COVID-19-Pandemie und die Maßnahmen zu deren Eindämmung verändern das Leben aller Menschen ad hoc. Bürger_innen werden zur Kontaktreduzierung angehalten, Institutionen des öffentlichen Lebens werden geschlossen – ihr Betrieb wird weitestgehend auf digitale Formate umgestellt. „Home“-Office, -Schooling, -Learning … kennzeichnen den neuen Alltag. Das gesellschaftliche Leben spielt sich plötzlich komplett digital und „at home“ ab. Auch Bildungseinrichtungen sind gefordert, der Situation entsprechende Formate zu entwickeln. Hochschulen werden teilweise ganz geschlossen oder sind nur noch für Personal, nicht aber für Studierende, zugänglich, Lehrveranstaltungen werden bundesweit zeitweise beinahe vollständig in digitalen Formaten durchgeführt und relevante hochschulische Infrastruktur (wie z. B. Bibliotheken, Arbeitsräume, Mensen) ist nur noch begrenzt bzw. unter bestimmten Voraussetzungen nutzbar (HRK 2022).

Mit diesen Einschränkungen verändert sich die hochschulische Organisation wie auch der Studierendenalltag grundlegend. Nach fast zwei Jahren der Pandemie rückt 2022 die Frage, welche Bedeutung diese Veränderungen auch für die langfristige bzw. zukünftige Gestaltung des Studierens generell sowie von Studiengängen haben könnte, zunehmend in den Fokus. Vorrangig wird dabei auf den Lehrbetrieb Bezug genommen und gefragt, wie der durch die Pandemie ausgelöste zwangsweise Digitalisierungsschub auch für die zukünftige Gestaltung von Lehre relevant sein könnte (vgl. u. a. Dittler und Kreidl 2021; Lübcke et al. 2022; Horstmann 2022). Die Beibehaltung digitalisierter und digital gestützter Lehrformate, ein stärkerer technisch-didaktischer Support für Lehrende, der Ausbau und die Aufrechterhaltung der technischen Infrastruktur sowie eine dauerhafte Mischung von Präsenz- und Onlinelehre sind dabei einige der thematisierten Aspekte (Lübcke et al. 2022; Horstmann 2022), deren Umsetzung jedoch als in hohem Maße von der Dauerhaftigkeit finanzieller Ressourcen abhängig eingeschätzt wird (Goertz und Hense 2021). Über den Lehrbetrieb hinausblickend zeigt sich gleichsam die hohe Bedeutung des sozialen Lebens am Campus, das auch in der zukünftigen Gestaltung von Studiengängen daher relevant bleiben muss (Traus et al. 2020; Besa et al. 2021).

## Stu.diCo: Studierendenbefragung im Kontext der Corona-Pandemie

Mitten in diese Zeit fallen die Erhebungen Stu.diCo I und II. Sie verfolgen den Anspruch, auf die bis dato zu wenig thematisierte Situation der Studierenden aufmerksam zu machen und ihre Perspektive in die politischen Diskurse einzubringen (Traus et al. 2020). Dabei sollte neben der Lehr- bzw. Studienorganisation im engeren Sinn besonders auch die Studierendensituation im weiteren Sinn erfasst werden. Neben geschlossenen Skalen u. a. zur psycho-sozialen Situation der Studierenden, ihrem Belastungserleben, ihren Unterstützungsressourcen und ihren Mitbestimmungsmöglichkeiten wurden dabei auch offene Fragen integriert, um die Perspektiven der Studierenden zu erfassen. So wurde beispielsweise die in diesem Beitrag fokussierte Frage danach, was auch in Zukunft für die Hochschulen erhalten bleiben soll, in der Stu.diCo II Erhebung im Sommersemester 2021 in Form eines Freitextfeldes gestellt.

Der Link zur Befragung^1^ wurde über verschiedene Netzwerke gestreut mit der Bitte um Weiterleitung an Studierende bzw. um Teilnahme (sog. Snowball-Sampling, Gabler 1992). Es handelt sich damit um eine selbstselektive Zufallsstichprobe. Insgesamt bezieht sich der bereinigte Datensatz auf *N* = 2527 Fälle. In die nachfolgenden Analysen sind die Daten derjenigen Befragten eingeflossen, die die Studienrichtung „Erziehungswissenschaft/Bildungswissenschaft“ ausgewählt haben (*n* = 405) sowie diejenigen, die eine „andere“ Studienrichtung ausgewählt und in das entsprechende Freitextfeld Studiengänge aus dem Bereich der Sozialpädagogik/Sozialen Arbeit eingetragen haben (z. B. Religions- und Gemeindepädagogik & Soziale Arbeit, Sozial- und Organisationspädagogik, *n* = 143). Der Altersdurchschnitt dieser Befragten lag bei 24,27 Jahren und damit etwas über dem Altersdurchschnitt der Gesamtstichprobe (M = 24,07 Jahre) und der weitaus überwiegende Teil war weiblich (86,1 %) (vgl. Tab. 1). Etwas mehr als ein Drittel der Befragten hat zwischen dem Sommersemester 2020 und dem Sommersemester 2021 ihr Studium begonnen und kennt damit nur das Studium unter den Bedingungen der Corona-Pandemie.

**Table 1:** 

*N*	548
Durchschnittsalter der Befragten	24,27 Jahre
Geschlecht	86,1 % Weiblich 13,1 % Männlich 0,5 % Divers 0,2 % keine dieser Kategorien
Studienbeginn	62,5 % vor dem Sommersemester 2020 37,5 % nach dem Sommersemester 2020

## Was von Studierenden besonders vermisst wurde …

**Figure 1:**
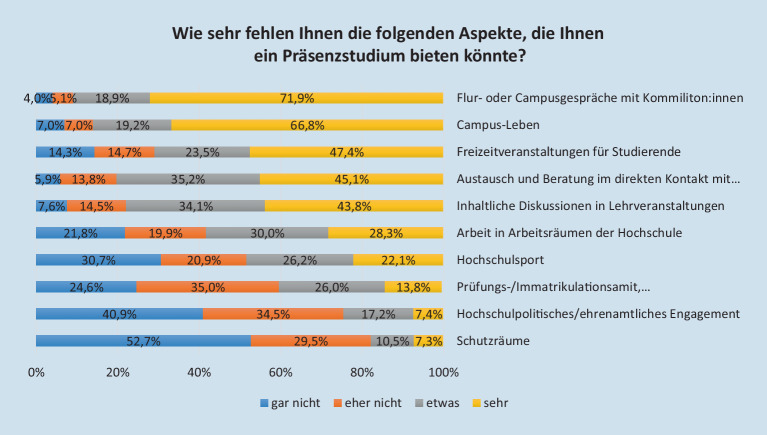

Danach gefragt, wie sehr den Studierenden bestimmte Aspekte fehlen, die ihnen ein Präsenzstudium bieten könnte, zeigt sich, dass insbesondere der direkte Kontakt zu Kommiliton_innen und das Campusleben (wie gemeinsames Essen in der Mensa) einem Großteil der Studierenden fehlt (vgl. Abb. 1). Auch Freizeitveranstaltungen für Studierende fehlen vielen Studierenden etwas/sehr. Die soziale Komponente des Studiums stellt demnach für die Studierenden einen zentralen Aspekt dar. Dies zeigten bereits die Ergebnisse der Studi.Co I Befragung. 79 % der Befragten gaben dort an, das Campusleben zu vermissen und 85,4 % fehlte der Kontakt zu anderen Studierenden. Daneben werden, wie in der ersten Befragung im Sommersemester 2020, im Sommer 2021 auch der inhaltliche Austausch in Seminaren sowie der persönliche Kontakt zu den Lehrenden von einem überwiegenden Anteil der Befragten (sehr/etwas) vermisst.

Die Bedeutung der sozialen Kontakte für die Studierenden zeigt sich auch in den Ergebnissen zu der Frage nach Nachteilen im digitalen Semester. So wählten 82,5 % der Befragten die Option „dass kein direkter Kontakt zu anderen besteht“ als einen der Top-3-Nachteile des digitalen Semesters. Diese Option wurde damit mit Abstand am häufigsten gewählt.

## … und was erhalten bleiben sollte

Von den 548 Befragten, deren Antworten Basis dieses Beitrags sind, haben 360 die Möglichkeit genutzt, in einem Freitextfeld auf die Frage zu antworten, was von den (zum damaligen Zeitpunkt) aktuellen Formaten an der Hochschule in Zukunft beibehalten werden sollte. Anhand der Freitexte wurde induktiv, also aus den Antworten heraus, ein Kategoriensystem gebildet. Viele Antworten umfassten mehrere Aspekte, sodass insgesamt 571 Kodierungen vorgenommen werden konnten. Das am häufigsten genannte Thema ist die (Digitalisierung der) Lehre. Daneben spielen auch der Aspekt Flexibilität/Vereinbarkeit, die Themen Studienorganisation und -bedingungen sowie Uni-Infrastrukturen eine zentrale Rolle. Die Ergebnisse aus diesen Kategorien werden daher im Folgenden näher dargestellt.

Die *Digitalisierung der Lehre* stellt in den Freitextantworten die zentrale Kategorie dar und wird in verschiedensten Aspekten angesprochen – dieses wurde im Kategoriensystem mittels Subkategorien abgebildet. Die mit Abstand meisten Nennungen lassen sich der Subkategorie *Veranstaltungsformate* zuordnen. Geäußert wird hier häufig der Wunsch nach hybriden (*n* = 53) sowie asynchronen (*n* = 93) Formaten. Hierzu passt auch der Wunsch nach einem Wechsel aus Präsenz- und Digitalangebot. Die Wahl des Veranstaltungsformats wird dabei nicht selten mit der Art der Veranstaltung verbunden. So werden von einigen Studierenden explizit Theorie vermittelnde, frontale Veranstaltungen mit vielen Teilnehmer_innen (= Vorlesungen) als Gegenstück zu interaktiven Veranstaltungen mit Austausch und Diskussion (= Seminare) in digitalem Format, meist als Aufzeichnung, gewünscht. Der Wunsch nach der Aufzeichnung von Veranstaltungen wird mit der Möglichkeit, die Veranstaltungen im gewünschten Tempo, mit Unterbrechung, mehrfach oder auch zum gewünschten Zeitpunkt anzusehen, begründet. Erwähnenswert ist zudem der Wunsch nach der weiteren Nutzung bzw. der Zurverfügungstellung digitaler Tools, wie Videokonferenztools oder auch Lernplattformen. Nur wenige Stimmen sprechen sich für die Rückkehr zur absoluten Präsenzlehre (*n* = 9) aus.

Auch in der Kategorie *Flexibilität/Verfügbarkeit* wurden Subkategorien zur weiteren Unterteilung eingesetzt. So wird der Wunsch nach an einer flexiblen Gestaltung des Studiums vor allem mit der Vereinbarkeit von *(Wohn‑)Ort, Beruf und Job, Familie/Kindern* sowie *Gesundheit* begründet. Mit Gesundheit ist dabei meist die Teilnahme an den Veranstaltungen von zu Hause bzw. das Nachholen der Veranstaltungen trotz Krankheit verbunden. Dieser Punkt spiegelt sich auch in der Kategorie Studienorganisation und -bedingungen wieder. Hier werden *digitale Formate für Prüfungsanmeldungen, Klausuren sowie Studien- und Prüfungsleistungen*, das Angebot *digitaler Sprechstunden* sowie die *Abschaffung der Anwesenheitspflicht* als Aspekte genannt, die aus Sicht der Studierenden beibehalten werden sollten.

Auch im Hinblick auf die *Hochschulinfrastrukturen* zeigt sich der Wunsch nach mehr Flexibilität. Hier wird vermehrt der digitale Zugriff auf *Literatur* genannt. Aber auch die Abwicklung *bürokratischer Kommunikation, Informationsvermittlung* und *Beratungsangebote *auf digitalem Wege wird gewünscht.

Ein eher kleiner Teil der Aussagen der Studierenden lässt sich unter der Kategorie *sozialen Aspekte *(*n* = 14) zusammenfassen – hier wird Hochschule konkret als sozialer Ort adressiert. Dabei wird zum einen die Relevanz von Begegnungen (face-to-face) – sowohl unter Studierenden als auch zwischen Studierenden und Lehrenden – hervorgehoben. Zum anderen wird der Wunsch nach Interesse und Verständnis für die verschiedenen Lebenssituationen von Studierenden im Kontext Hochschule geäußert. Dass bei der Frage danach „Was soll […] beibehalten bleiben?“ nur von wenigen der Befragten „soziale Aspekte“ genannt werden, obwohl die hohe Bedeutung sozialer Kontakte an anderer Stelle zum Ausdruck gebracht wurde, weist erneut darauf hin, dass Hochschule als sozialer Ort im Zuge der Umstellung auf digitale Formate im Zuge der Corona-Pandemie aus dem Blick geraten ist. Anscheinend gibt es hier wenig „Soziales“, das beibehalten bleiben könnte – face-to-face Kontakte lassen sich nicht einfach durch digitale Formate ersetzen.

Beispielhaft für die dargestellten Inhalte – und diese miteinander in Beziehung setzend – der Freitextantworten zu der Frage „Aus Ihren Erfahrungen aus dem digitalen Studieren heraus: Was sollte Ihrer Meinung nach aus den aktuellen Formaten an der Hochschule in Zukunft beibehalten bleiben?“ dient das folgende Zitat: *„Hybrider Zugang zu Vorlesungen und Seminaren, sowohl in Präsenz anbieten als auch digital oder z.* *B. das Mitfilmen von Vorlesungen und anschließende Bereitstellung, sodass man auch teilnehmen kann, wenn der Fahrtweg zu lang ist oder man sich als Elternteil um die Kinder kümmern muss. Digitale Zugangsmöglichkeiten schaffen den Vorteil der flexiblen Gestaltung des eigenen Lernens (unabhängig von Zeit und Ort), wichtig ist dabei, nicht den persönlichen Kontakt und die soziale Einbindung zu vernachlässigen!“*

## Fazit

Die diesem Schwerpunkt zu Grunde liegenden Fragen wurden in diesem Beitrag anhand ausgewählter Ergebnisse aus der Studie Stu.diCo II mit Blick auf die didaktische Ausgestaltung des Studiums „visionärisch“ beantwortet. Mit einem Blick auf die Vision „Studieren 2035“ und den mittels Stu.diCo abgebildeten Jetzt-Zustand wird deutlich, dass Hochschulen einen langen Weg vor sich haben, falls sie sich den abgebildeten Bedarfen annehmen und aus den neuen Erfahrungen, die die Corona-Pandemie mit sich gebracht haben, lernen wollen. Sie sind gefordert, den passenden Rahmen zur Verfügung zu stellen, größtmögliche Flexibilität zu ermöglichen, die passende Infrastruktur vor zu halten und gleichzeitig Orte des Zusammenkommens zu gestalten. Auch wenn durchaus kritisch hinterfragt werden darf, ob eine Realisierung der eingangs formulierten Vision tatsächlich wünschenswert wäre, bieten die Erkenntnisse aus der Zeit der Corona-Pandemie sicherlich eine Grundlage zum Weiterdenken und -entwickeln hochschulischer Strukturen.

## Footnotes

1 Durchführung über www.soscisurvey.de.
